# Prediction of hierarchical time series using structured regularization and its application to artificial neural networks

**DOI:** 10.1371/journal.pone.0242099

**Published:** 2020-11-12

**Authors:** Tomokaze Shiratori, Ken Kobayashi, Yuichi Takano

**Affiliations:** 1 Graduate School of Systems and Information Engineering, University of Tsukuba, Tsukuba, Ibaraki, Japan; 2 Artificial Intelligence Laboratory, Fujitsu Laboratories Ltd., Kawasaki, Kanagawa, Japan; 3 Faculty of Engineering, Information and Systems, University of Tsukuba, Tsukuba, Ibaraki, Japan; Utrecht University, NETHERLANDS

## Abstract

This paper discusses the prediction of hierarchical time series, where each upper-level time series is calculated by summing appropriate lower-level time series. Forecasts for such hierarchical time series should be coherent, meaning that the forecast for an upper-level time series equals the sum of forecasts for corresponding lower-level time series. Previous methods for making coherent forecasts consist of two phases: first computing base (incoherent) forecasts and then reconciling those forecasts based on their inherent hierarchical structure. To improve time series predictions, we propose a structured regularization method for completing both phases simultaneously. The proposed method is based on a prediction model for bottom-level time series and uses a structured regularization term to incorporate upper-level forecasts into the prediction model. We also develop a backpropagation algorithm specialized for applying our method to artificial neural networks for time series prediction. Experimental results using synthetic and real-world datasets demonstrate that our method is comparable in terms of prediction accuracy and computational efficiency to other methods for time series prediction.

## Introduction

Multivariate time series data often have a hierarchical (tree) structure in which each upper-level time series is calculated by summing appropriate lower-level time series. For instance, numbers of tourists are usually counted on a regional basis, such as sites, cities, regions, or countries [1]. Similarly, many companies require regionally aggregated forecasts to support resource allocation decisions [2]. Product demand is often analyzed by category to reduce the overall forecasting burden [3].

Forecasts for such hierarchical time series should be *coherent*, meaning that the forecast for an upper-level time series equals the sum of forecasts for corresponding lower-level time series [4, 5]. Smoothing methods such as the moving average and exponential smoothing are widely used in academia and industry for time series predictions [6, 7]. Although these methods provide coherent forecasts for hierarchical time series, they have low accuracy, especially for rapidly changing time series.

Another common approach for making coherent forecasts is the use of bottom-up and top-down methods [3, 8–10]. These methods first develop base forecasts by separately predicting each time series and then reconcile those base forecasts based on their inherent hierarchical structure. The bottom-up method calculates base forecasts for bottom-level time series and then aggregates them for upper-level time series. In contrast, the top-down method calculates base forecasts only for a root (total) time series and then disaggregates them according to historical proportions of lower-level time series. Park and Nassar [11] considered a hierarchical Bayesian dynamic proportions model for the top-down method to disaggregate upper-level forecasts sequentially. The middle-out method calculates base forecasts for intermediate-level time series and then applies the bottom-up and top-down methods to make upper- and lower-level forecasts. However, the bottom-up method often accumulates prediction errors as the time series level rises, and the top-down method cannot exploit detailed information about lower-level time series. Notably, when base forecasts are unbiased, only the bottom-up method gives unbiased forecasts [12].

Hyndman et al. [12] proposed a linear regression approach to optimal base forecasts by the bottom-up method. This forecast reconciliation method worked well for predicting tourism demand [1] and monthly inflation [13], and this approach can be extended to hierarchical and grouped time series [14]. van Erven and Cugliari [15] devised a game-theoretically optimal reconciliation method. Regularized regression models have also been employed to deal with high-dimensional time series [16, 17]. Wickramasuriya et al. [5] devised a sophisticated method for optimal forecast reconciliation through trace minimization. Their experimental results showed that this trace minimization method performed very well with synthetic and real-world datasets. Note, however, that all of these forecast reconciliation methods consist of two phases: first computing base forecasts and then reconciling those forecasts based on a hierarchical structure. This study aimed to produce better time series predictions by simultaneously completing these two phases.

Structured regularization uses inherent structural relations among explanatory variables to construct a statistical model [18–20]. Various regularization methods have been proposed for multivariate time series [21, 22], hierarchical explanatory variables [23–26], and artificial neural networks [27]. Prediction of multivariate time series is related to multitask learning, which shares useful information among related tasks to enhance the prediction performance for all tasks [28, 29]. Tailored regularization methods have been developed for multitask learning [30, 31] and applied to artificial neural networks [32]. To the best of our knowledge, however, no prior studies have applied structured regularization methods to predictions of hierarchical time series.

In this study, we aimed to develop a structured regularization method that takes full advantage of hierarchical structure for better time series predictions. Our method is based on a prediction model for bottom-level time series and uses a structured regularization term to incorporate upper-level forecasts into the prediction model. This study particularly focused on applying our method to artificial neural networks, which have been effectively used in time series prediction [33–38]. We developed a backpropagation algorithm specialized for our structured regularization model based on artificial neural networks. Experiments involving the application of our method to synthetic and real-world datasets demonstrated that our method was comparable in terms of prediction accuracy and computational efficiency to other methods that develop coherent forecasts for hierarchical time series.

## Methods

This section starts with a brief review of forecasts for hierarchical time series. For such time series, we present our structured regularization model and its application to artificial neural networks. A backpropagation algorithm is also described for artificial neural networks with structured regularization.

### Forecasts for hierarchical time series

**Fig 1:**
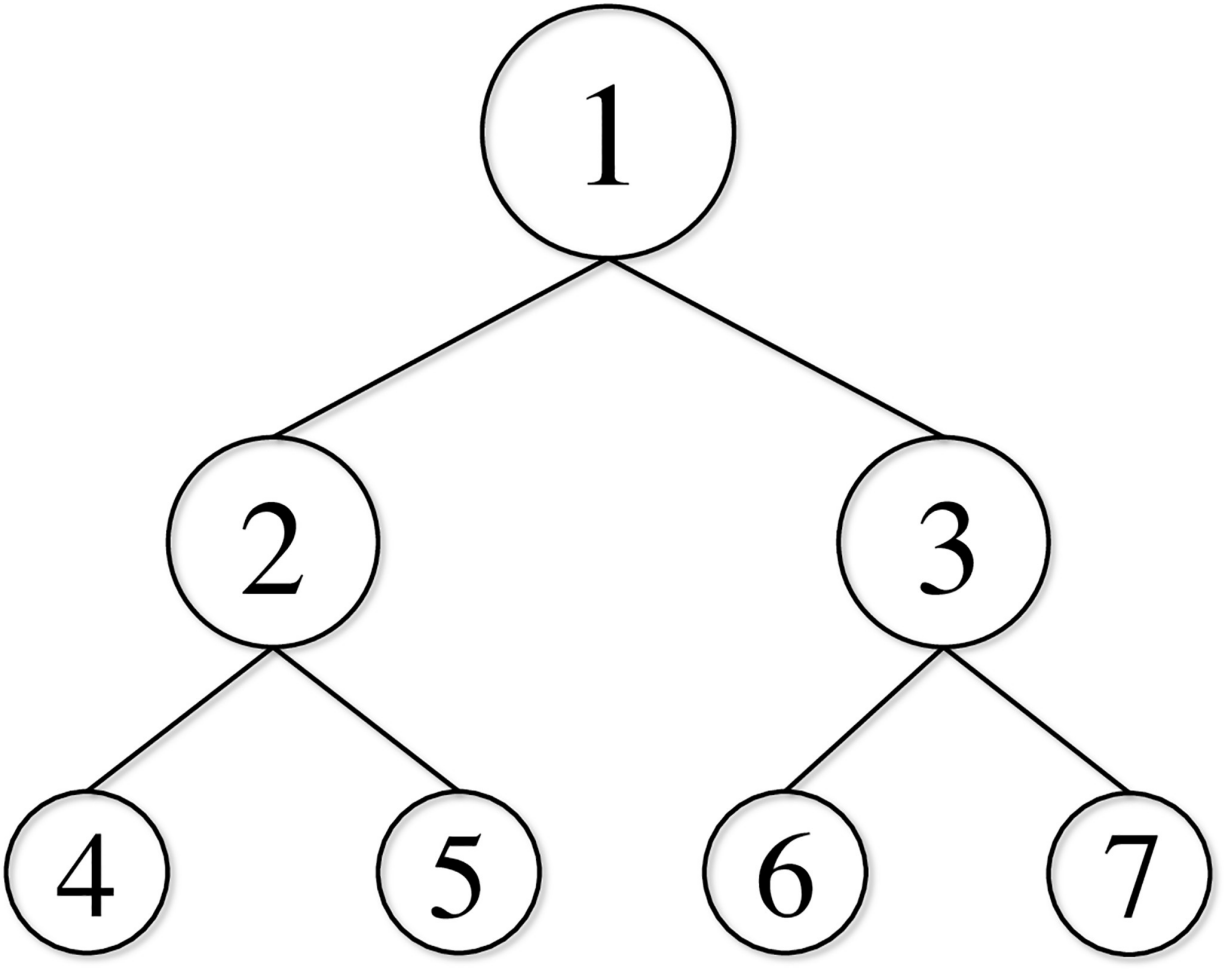
Two-level hierarchical structure with |*N*| = 7.

We address the prediction of multivariate time series where each series is represented as a node in a hierarchical (tree) structure. Let *y*_*it*_ be an observation of node *i* ∈ *N* at time *t* ∈ *T*, where *N* is the set of nodes, and *T* is the set of time points. For simplicity, we focus on two-level hierarchical structures. Fig 1 shows the example of a two-level hierarchical structure with |*N*| = 7 nodes, where | ⋅ | denotes the number of set elements. The nodes are classified as

N={1}∪M∪B,M={2,3},B={4,5,6,7},

where node 1 is the root (level-zero) node, and *M* and *B* are sets of mid-level (level-one) and bottom-level (level-two) nodes, respectively. The associated time series is characterized by the *aggregation constraint*

$$\begin{array}{c} \left\{ \begin{array}{c} \;y_{1t}=y_{4t}+y_{5t}+y_{6t}+y_{7t},\\ \;y_{2t}=y_{4t}+y_{5t},\\ \;y_{3t}=y_{6t}+y_{7t}, \end{array}\;\left(t\in T\right).\right. \end{array}$$ (1)

Each upper-level time series is thus calculated by summing the corresponding lower-level time series.

A hierarchical structure is represented by the *structure matrix* ***H*** ≔ (*h*_*ki*_)_(*k*,*i*)∈(*N*\*B*)×*B*_ as

$$\begin{array}{c} h_{ki}=\{\begin{array}{cc} 1 & \text{if}\;\text{node}\;k\;\text{is}\;\text{an}\;\text{ascendant}\;\text{of}\;\text{node}\;i,\\ 0 & \text{otherwise}, \end{array}\;\left(k\in N\backslash B,\;i\in B\right). \end{array}$$

We define the *summing matrix* as

$$S≔{\left(s_{ki}\right)}_{(k,i)\in N\times B}≔[\begin{array}{c} H\\ I_{\left|B\right|} \end{array}],$$

where ***I***_*n*_ is the identity matrix of size *n*. In Fig 1, we have

$$H=\left[\begin{array}{cccc} 1 & 1 & 1 & 1\\ 1 & 1 & 0 & 0\\ 0 & 0 & 1 & 1 \end{array}\right],\;S=[\begin{array}{cccc} 1 & 1 & 1 & 1\\ 1 & 1 & 0 & 0\\ 0 & 0 & 1 & 1\\ 1 & 0 & 0 & 0\\ 0 & 1 & 0 & 0\\ 0 & 0 & 1 & 0\\ 0 & 0 & 0 & 1 \end{array}].$$

Let ***y***_*t*_ ≔ (*y*_*it*_)_*i*∈*N*_ be a column vector comprising observations of all nodes at time *t* ∈ *T*. Similarly, for a node subset *A* ⊆ *N* we define $y_{t}^{A}≔{\left(y_{it}\right)}_{i\in A}$ as the observation vector of nodes *i* ∈ *A* at time *t* ∈ *T*. In Fig 1, we have

$$\begin{array}{cc} & y_{t}={\left(y_{1t},y_{2t},y_{3t},y_{4t},y_{5t},y_{6t},y_{7t}\right)}^{⊤},\\ & y_{t}^{M}={\left(y_{2t},y_{3t}\right)}^{⊤},\;y_{t}^{B}={\left(y_{4t},y_{5t},y_{6t},y_{7t}\right)}^{⊤}\;\left(t\in T\right). \end{array}$$

The aggregation constraint (1) is then expressed as

$$\begin{array}{c} y_{t}^{N\backslash B}=Hy_{t}^{B}\;\left(t\in T\right) \end{array}$$ (2)

or, equivalently,

$$\begin{array}{c} y_{t}=Sy_{t}^{B}\;\left(t\in T\right). \end{array}$$ (3)

Let ${\hat{y}}_{t}≔{\left({\hat{y}}_{it}\right)}_{i\in N}$ be a column vector comprising *base forecasts* at time *t* ∈ *T*. Note that the base forecasts are calculated separately for each node *i* ∈ *N*, so they do not satisfy the aggregation constraint (2). For a node subset *A* ⊆ *N*, we define ${\hat{y}}_{t}^{A}≔{\left({\hat{y}}_{it}\right)}_{i\in A}$ at time *t* ∈ *T*. Such base forecasts can be converted into *coherent forecasts* satisfying the aggregation constraint (2) by using the *reconciliation matrix* ***P*** ≔ (*p*_*ij*_)_(*i*,*j*)∈*B*×*N*_. Specifically, we develop bottom-level forecasts ${\tilde{y}}_{t}^{B}=P{\hat{y}}_{t}$ and use the aggregation constraint (3) to obtain coherent forecasts, as

$$\begin{array}{c} {\tilde{y}}_{t}=SP{\hat{y}}_{t}\;\left(t\in T\right). \end{array}$$ (4)

A typical example of a reconciliation matrix is

$$P=[O_{\left|B|\times |N\backslash B\right|},\;I_{\left|B\right|}],$$

where ***O***_*m*×*n*_ is an *m* × *n* zero matrix. This leads to the bottom-up method

$$\begin{array}{c} {\tilde{y}}_{t}=S{\hat{y}}_{t}^{B}\;\left(t\in T\right). \end{array}$$ (5)

Another example is

$$P=[p,\;O_{\left|B|\times |N\backslash \{1\}\right|}],$$

where ***p*** = (*p*_*i*_)_*i*∈*B*_ is a column vector comprising historical proportions of bottom-level time series. This results in the top-down method

$${\tilde{y}}_{t}=S\left({\hat{y}}_{1t}p\right)\;\left(t\in T\right).$$

In this manner, we can make coherent forecasts from various reconciliation matrices. The condition ***SPS*** = ***S*** is proven to ensure that when base forecasts are unbiased, the resultant coherent forecasts (4) are also unbiased [12]. This condition is also known to be fulfilled only by the bottom-up method [12].

### Forecast reconciliation methods

Hyndman et al. [12] introduced the following linear regression model for given base forecasts:

$${\hat{y}}_{t}=S\beta_{t}+\varepsilon_{t}\;\left(t\in T\right),$$

where ***β***_*t*_ ≔ (*β*_*it*_)_*i*∈*B*_ is a column vector of bottom-level estimates, and *ε*_*t*_ ≔ (*ε*_*it*_)_*i*∈*B*_ is a column vector of errors having zero mean and covariance matrix var(*ε*_*t*_) ≔ Σ_*t*_. The bottom-up method (5) with ${\hat{y}}_{t}^{B}=\beta_{t}$ is then used to makes coherent forecasts.

If the base forecasts are unbiased and the covariance matrix Σ_*t*_ is known, the generalized least-squares estimation yields the minimum variance unbiased estimate of *β*_*t*_. However, the covariance matrix Σ_*t*_ is nonidentifiable and therefore impossible to estimate [5].

In contrast, Wickramasuriya et al. [5] focused on differences between observations and coherent forecasts (4),

$$e_{t}≔y_{t}-{\tilde{y}}_{t}=y_{t}-SP{\hat{y}}_{t}\;\left(t\in T\right).$$

The associated covariance matrix is derived as

$$\begin{array}{c} \text{var}\left(e_{t}\right)=SPW_{t}P^{⊤}S^{⊤}\;\left(t\in T\right), \end{array}$$ (6)

where $W_{t}≔E\left[\left(y_{t}-{\hat{y}}_{t}\right){\left(y_{t}-{\hat{y}}_{t}\right)}^{⊤}\right]$ is the covariance matrix of base forecasts. The trace of the covariance matrix (6) is minimized subject to the unbiasedness condition ***SPS*** = ***S***. This yields the optimal reconciliation matrix

$$P={\left(S^{⊤}W_{t}^{-1}S\right)}^{-1}S^{⊤}W_{t}^{-1},$$

and coherent forecasts (4) are given by

$$\begin{array}{c} {\tilde{y}}_{t}=S{\left(S^{⊤}W_{t}^{-1}S\right)}^{-1}S^{⊤}W_{t}^{-1}{\hat{y}}_{t}\;\left(t\in T\right). \end{array}$$ (7)

See Wickramasuriya et al. [5] for the full details.

Note, however, that in these forecast reconciliation methods, base forecasts are first determined regardless of the underlying hierarchical structure, then those forecasts are corrected based on the hierarchical structure. In contrast, our proposal is a structured regularization model that directly computes high-quality forecasts based on the hierarchical structure.

### Structured regularization model

We consider a prediction model for bottom-level time series. Its predictive value is denoted by the column vector ${\hat{y}}_{t}^{B}\left(\Theta \right)≔{\left({\hat{y}}_{it}\left(\Theta \right)\right)}_{i\in B}$, where **Θ** is a tuple of model parameters. As an example, the first-order vector autoregressive model is represented as

$${\hat{y}}_{it}\left(\Theta \right)=\sum_{j\in B}\theta_{ij}y_{j,t-1}\;\left(i\in B,\;t\in T\right),$$

where **Θ** = (*θ*_*ij*_)_(*i*,*j*)∈*B*×*B*_.

The residual sum of squares for bottom-level time series is given by

$$\begin{array}{c} \sum_{t\in T}{\left\|y_{t}^{B}-{\hat{y}}_{t}^{B}\left(\Theta \right)\right\|}_{2}^{2}=\sum_{t\in T}\sum_{i\in B}{\left(y_{it}-{\hat{y}}_{it}\left(\Theta \right)\right)}^{2}. \end{array}$$ (8)

We also introduce a structured regularization term that quantifies the error for upper-level time series based on the hierarchical structure. Let **Λ** ≔ Diag(**λ**) be a diagonal matrix of regularization parameters, where **λ** ≔ (λ_*i*_)_*i*∈*N*\*B*_ is a vector of its diagonal entries. Then, we construct a structured regularization term based on the aggregation constraint (2) as

$$\begin{array}{c} \sum_{t\in T}{\left\|\Lambda \left(y_{t}^{N\backslash B}-H{\hat{y}}_{t}^{B}\left(\Theta \right)\right)\right\|}_{2}^{2}. \end{array}$$ (9)

Minimizing this term aids in correcting bottom-level forecasts, thus improving the upper-level forecasts.

Adding the regularization term (9) to the residual sum of squares (8) yields the objective function *E*(**Θ**) to be minimized. Consequently, our structured regularization model is posed as

$$\begin{array}{c} \Theta^{*}\in \underset{\Theta }{\text{argmin}}\{E\left(\Theta \right)≔\frac{1}{2}\sum_{t\in T}\|y_{t}^{B}-{\hat{y}}_{t}^{B}{\left(\Theta \right)\|}_{2}^{2}+\frac{1}{2}\sum_{t\in T}{\left\|\Lambda \left(y_{t}^{N\backslash B}-H{\hat{y}}_{t}^{B}\left(\Theta \right)\right)\right\|}_{2}^{2}\}. \end{array}$$ (10)

Here, matrix **Λ** adjusts the tradeoff between minimizing the error term (8) for bottom-level times series and minimizing the error term (9) for upper-level time series. In the experiments section, we set its diagonal entries as

$$\begin{array}{c} \lambda_{i}=\{\begin{array}{cc} \lambda_{1} & (i=1),\\ \lambda_{M} & (i\in M), \end{array} \end{array}$$ (11)

where λ_1_ and λ_*M*_ are regularization parameters for root and mid-level time series, respectively.

After solving the structured regularization model (10), we use the bottom-up method (5) to obtain coherent forecasts

$${\tilde{y}}_{t}=S{\hat{y}}_{t}^{B}\left(\Theta^{*}\right).$$

Our structured regularization model based on the bottom-up method may not work well when upper-level time series are easier to predict than bottom-level time series. To remedy this situation, we can adopt a methodology proposed by Panagiotelis et al. [39], where the summing matrix is redefined by replacing a bottom-level time series with an upper-level time series.

### Application to artificial neural networks

**Fig 2:**
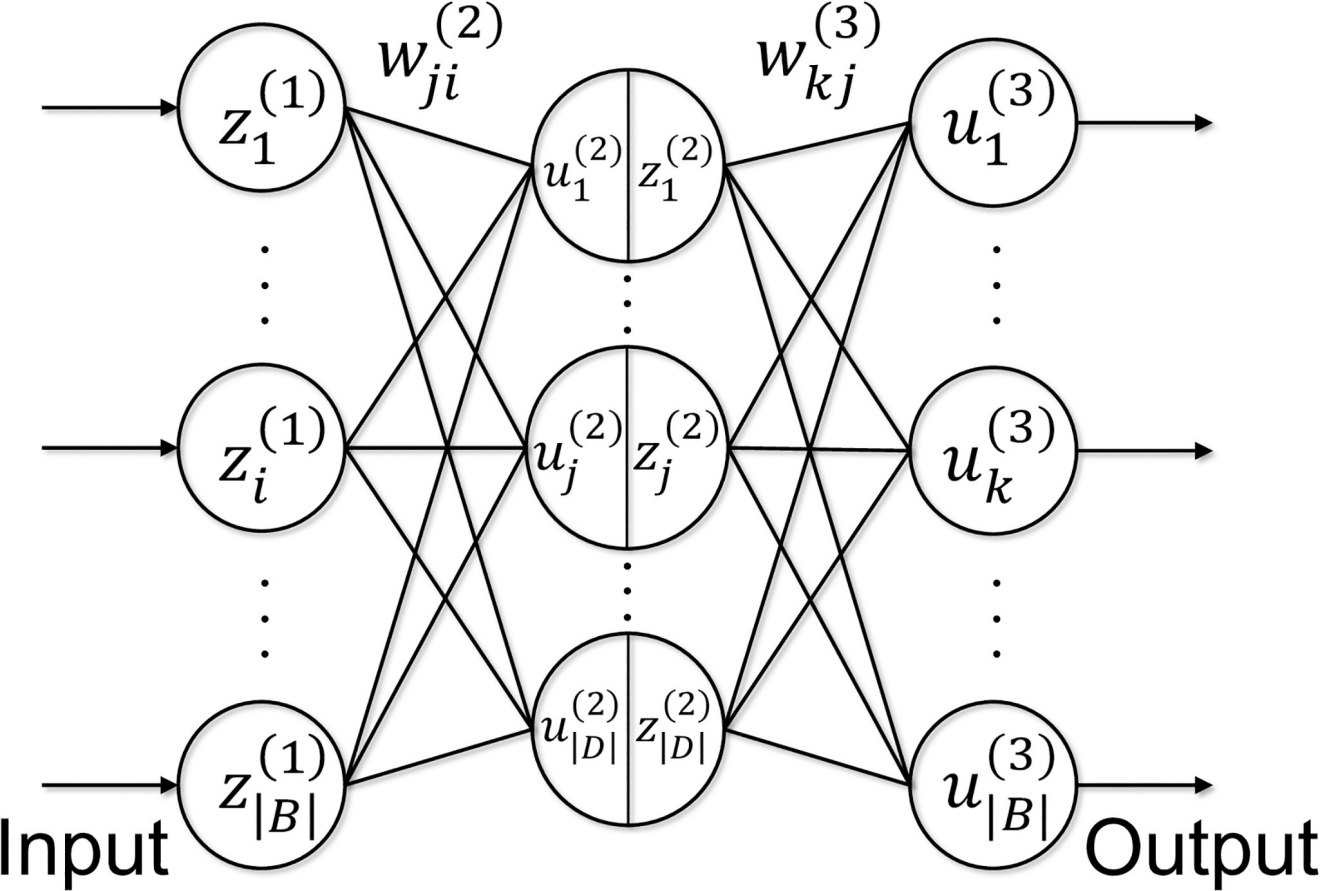
Network diagram for a two-layer neural network.

This study focused on application of our structured regularization model (10) to artificial neural networks for time series prediction; see Bishop [40] and Goodfellow et al. [41] for general descriptions of artificial neural networks. For simplicity, we consider a two-layer neural network like the one shown in Fig 2, where the input vector $z^{\left(1\right)}≔{\left(z_{i}^{\left(1\right)}\right)}_{i\in B}$ is defined as

$$z_{i}^{\left(1\right)}=y_{i,t-1}\;\left(i\in B\right).$$

First, we calculate the vector $u^{\left(2\right)}≔{\left(u_{j}^{\left(2\right)}\right)}_{j\in D}$ as the weighted sum of the input entries

$$\begin{array}{c} u_{j}^{\left(2\right)}=\sum_{i\in B}w_{ji}^{\left(2\right)}z_{i}^{\left(1\right)}\;\left(j\in D\right), \end{array}$$ (12)

where $W^{\left(2\right)}≔{\left(w_{ji}^{\left(2\right)}\right)}_{(j,i)\in D\times B}$ is a weight matrix to be estimated. This vector ***u***^(2)^ is transferred from the input units to *hidden units*, as shown in Fig 2.

Next, we generate the vector $z^{\left(2\right)}≔{\left(z_{j}^{\left(2\right)}\right)}_{j\in D}$ by nonlinear *activation functions* as

$$z_{j}^{\left(2\right)}=f\left(u_{j}^{\left(2\right)}\right)\;\left(j\in D\right).$$

Typical examples of activation functions include the logistic sigmoid function

$$\begin{array}{c} f\left(u\right)=\frac{1}{1+\exp(-u)} \end{array}$$ (13)

and the rectified linear function

f(u)=max{u,0}.

The vector ***z***^(2)^ is transferred from the hidden units to the output units as shown in Fig 2.

Finally, we calculate the vector $u^{\left(3\right)}≔{\left(u_{k}^{\left(3\right)}\right)}_{k\in B}$ as the weighted sum of the output entries from the hidden units as

$$\begin{array}{c} u_{k}^{\left(3\right)}=\sum_{j\in D}w_{kj}^{\left(3\right)}z_{j}^{\left(2\right)}=\sum_{j\in D}w_{kj}^{\left(3\right)}f\left(u_{j}^{\left(2\right)}\right)\;\left(k\in B\right), \end{array}$$ (14)

where $W^{\left(3\right)}≔{\left(w_{kj}^{\left(3\right)}\right)}_{(k,j)\in B\times D}$ is a weight matrix to be estimated.

This process is summarized as

$$\begin{array}{c} z^{\left(1\right)}=y_{t-1}^{B},\;u^{\left(2\right)}=W^{\left(2\right)}z^{\left(1\right)},\;z^{\left(2\right)}=f\left(u^{\left(2\right)}\right),\;u^{\left(3\right)}=W^{\left(3\right)}z^{\left(2\right)}, \end{array}$$ (15)

where the tuple of model parameters is

$$\Theta =(W^{\left(2\right)},W^{\left(3\right)}).$$

This neural network outputs ${\hat{y}}_{t}^{B}(\Theta )=u^{\left(3\right)}$ as a vector of predictive values.

### Backpropagation algorithm

We develop a backpropagation algorithm specialized for training artificial neural networks in our structured regularization model (10); see Bishop [40] and Goodfellow et al. [41] for overviews of backpropagation algorithms. Our algorithm sequentially minimizes the following error function for time *t* ∈ *T*:

$$\begin{array}{cc} & E_{t}\left(\Theta \right)≔\frac{1}{2}\|y_{t}^{B}-u^{\left(3\right)}{\|}_{2}^{2}+\frac{1}{2}{\left\|\Lambda \left(y_{t}^{N\backslash B}-Hu^{\left(3\right)}\right)\right\|}_{2}^{2}\;\left(t\in T\right). \end{array}$$ (16)

We first define vectors $\delta^{\left(2\right)}≔{\left(\delta_{j}^{\left(2\right)}\right)}_{j\in D}$ and $\delta^{\left(3\right)}≔{\left(\delta_{k}^{\left(3\right)}\right)}_{k\in B}$, which consist of partial derivatives of the error function (16) with respect to intermediate variables (12) and (14) as follows:

$$\begin{array}{c} \delta_{j}^{\left(2\right)}≔\frac{\partial E_{t}\left(\Theta \right)}{\partial u_{j}^{\left(2\right)}}\;\left(j\in D\right),\;\delta_{k}^{\left(3\right)}≔\frac{\partial E_{t}\left(\Theta \right)}{\partial u_{k}^{\left(3\right)}}\;\left(k\in B\right). \end{array}$$

From Eqs (12) and (14), the partial derivatives of the error function (16) can be calculated as

$$\begin{array}{c} \frac{\partial E_{t}\left(\Theta \right)}{\partial w_{ji}^{\left(2\right)}}=\frac{\partial E_{t}\left(\Theta \right)}{\partial u_{j}^{\left(2\right)}}\frac{\partial u_{j}^{\left(2\right)}}{\partial w_{ji}^{\left(2\right)}}=\delta_{j}^{\left(2\right)}z_{i}^{\left(1\right)}\;\left(i\in B,\;j\in D\right), \end{array}$$ (17)

$$\begin{array}{c} \frac{\partial E_{t}\left(\Theta \right)}{\partial w_{kj}^{\left(3\right)}}=\frac{\partial E_{t}\left(\Theta \right)}{\partial u_{k}^{\left(3\right)}}\frac{\partial u_{k}^{\left(3\right)}}{\partial w_{kj}^{\left(3\right)}}=\delta_{k}^{\left(3\right)}z_{j}^{\left(2\right)}\;\left(j\in D,\;k\in B\right). \end{array}$$ (18)

From Eq (14), we have

$$\frac{\partial u_{k}^{\left(3\right)}}{\partial u_{j}^{\left(2\right)}}=w_{kj}^{\left(3\right)}f^{\prime }\left(u_{j}^{\left(2\right)}\right)\;\left(j\in D,\;k\in B\right).$$

Therefore,

$$\begin{array}{c} \delta_{j}^{\left(2\right)}=\frac{\partial E_{t}\left(\Theta \right)}{\partial u_{j}^{\left(2\right)}}=\sum_{k\in B}\frac{\partial E_{t}\left(\Theta \right)}{\partial u_{k}^{\left(3\right)}}\frac{\partial u_{k}^{\left(3\right)}}{\partial u_{j}^{\left(2\right)}}=\sum_{k\in B}\delta_{k}^{\left(3\right)}w_{kj}^{\left(3\right)}f^{\prime }\left(u_{j}^{\left(2\right)}\right)\;\left(j\in D\right). \end{array}$$ (19)

It follows from Eq (16) that

$$\begin{array}{cc} \delta^{\left(3\right)}≔\frac{\partial E_{t}\left(\Theta \right)}{\partial u^{\left(3\right)}} & =-\left(y_{t}^{B}-u^{\left(3\right)}\right)-{\left(\Lambda H\right)}^{⊤}\Lambda \left(y_{t}^{N\backslash B}-Hu^{\left(3\right)}\right)\\ & =-\left(y_{t}^{B}-u^{\left(3\right)}\right)-H^{⊤}\Lambda^{2}\left(y_{t}^{N\backslash B}-Hu^{\left(3\right)}\right)\\ & =-\left[H^{⊤}\Lambda^{2},\;I_{\left|B\right|}\right]y_{t}+\left(I_{\left|B\right|}+H^{⊤}\Lambda^{2}H\right)u^{\left(3\right)}. \end{array}$$ (20)

Algorithm 1 summarizes our backpropagation algorithm.

**Algorithm 1** Backpropagation algorithm.

**Step 0 (Initialization)**: Let *η* > 0 be a step size and *ε* > 0 be a threshold for convergence. Set **Θ** = (***W***^(2)^, ***W***^(3)^) as initial parameter values, and *E* ← *E*(**Θ**) = ∑_*t*∈*T*_ *E*_*T*_(**Θ**) as an incumbent value of the objective function.

**Step 1 (Backpropagation)**: Repeat the following steps for all *t* ∈ *T*:

 **Step 1.1**: Compute ***z***^(1)^, ***u***^(2)^, ***z***^(2)^, and ***u***^(3)^ from Eq (15).

 **Step 1.2**: Compute ***δ***^(3)^ from Eq (20) and then ***δ***^(2)^ from Eq (19).

 **Step 1.3**: Compute the partial derivatives (17) and (18).

**Step 2 (Gradient Descent)**: Update the weight parameter values as

$$\{\begin{array}{l} w_{ji}^{\left(2\right)}\leftarrow w_{ji}^{\left(2\right)}-\eta \sum_{t\in T}\frac{\partial E_{i}(\Theta )}{\partial w_{ji}^{\left(2\right)}}\;(i\in B,j\in D),\\ w_{ji}^{\left(3\right)}\leftarrow w_{ji}^{\left(3\right)}-\eta \sum_{t\in T}\frac{\partial E_{i}(\Theta )}{\partial w_{ji}^{\left(3\right)}}\;(j\in D,k\in B), \end{array}$$

**Step 3(Termination Condition)**: If *E*(**Θ**) > (1 − *ε*)*E*, terminate the algorithm with **Θ** = (***W***^(2)^, ***W***^(3)^). Otherwise, set *E* ← *E*(**Θ**) and return to Step 1.

## Experimental results and discussion

**Fig 3:**
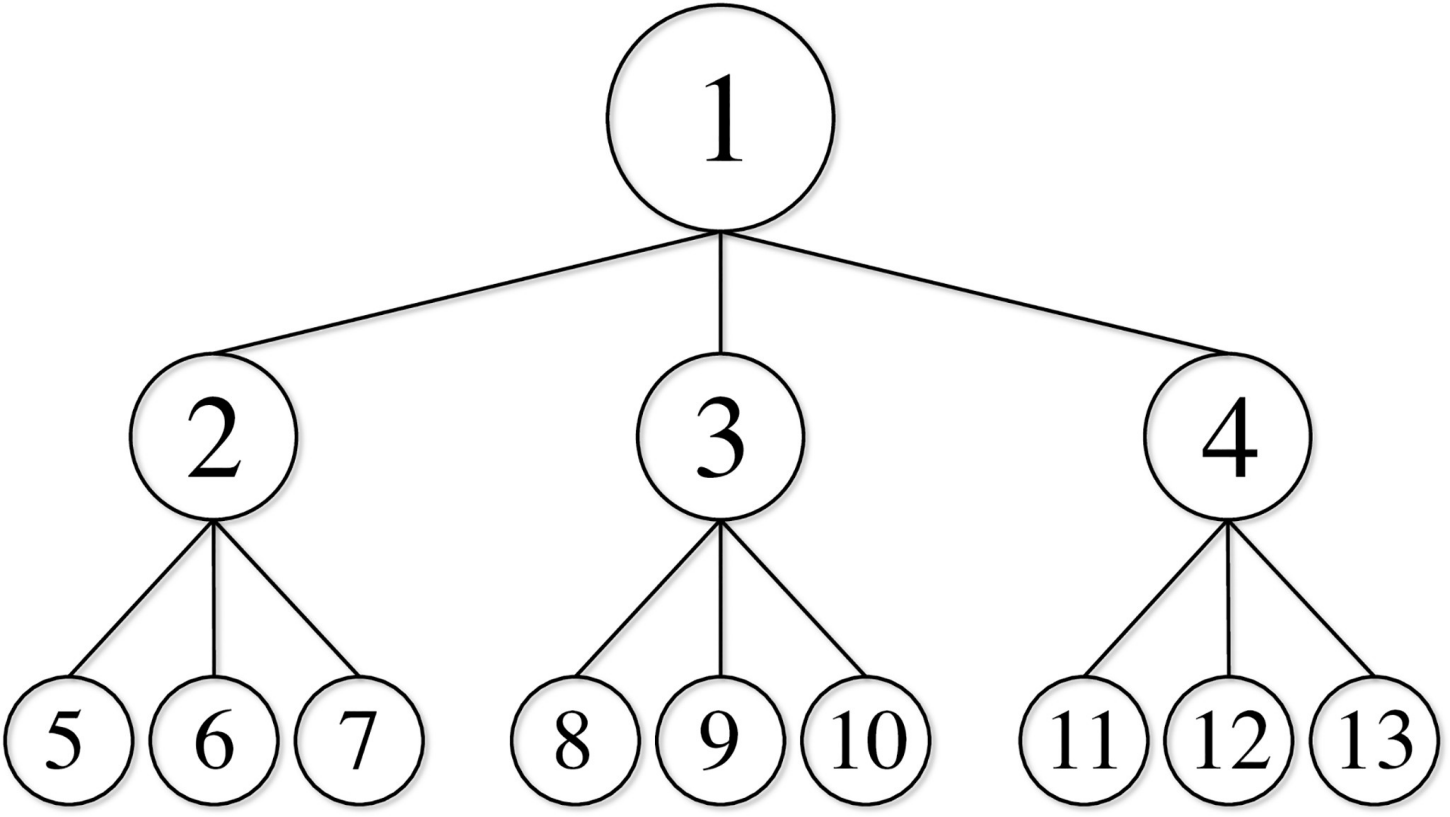
Two-level hierarchical structure with |*N*| = 13.

The experimental results reported in this section evaluate the effectiveness of our structured regularization model when applied to artificial neural networks. These experiments focused on the two-level hierarchical structure shown in Fig 3, where

N={1}∪M∪B,M={2,3,4},B={5,6,…,13}.

### Performance evaluation methodology

To evaluate out-of-sample prediction performance, we considered training and test periods of time series data, where the training period was used to train prediction models, and the test period was used to compute prediction errors in the trained models. We calculated the root-mean-squared error (RMSE) for each node *i* ∈ *N* during the test period $\hat{T}$ as

$$\text{RMSE}≔\sqrt{\frac{\sum_{t\in \hat{T}}{\left(y_{it}-{\tilde{y}}_{it}\right)}^{2}}{|\hat{T}|}}\;\left(i\in N\right).$$

We compared the performance of the following methods for time series prediction.

**MA**(*n*): moving average of the previous *n* values,
  $${\tilde{y}}_{it}=\frac{\sum_{k=1}^{n}y_{i,t-k}}{n}\;\left(i\in N,\;t\in T\right)$$
**ES**(*α*): exponential smoothing with a smoothing parameter *α* ∈ [0, 1],
  $${\tilde{y}}_{it}=\alpha y_{i,t-1}+\left(1-\alpha \right){\tilde{y}}_{i,t-1}\;\left(i\in N,\;t\in T\right)$$
**NN+BU**: bottom-up method (5) using artificial neural networks for base forecasts ${\hat{y}}_{t}^{B}$
**NN+MinT**: forecast reconciliation method (7) through the trace minimization (i.e., MinT(Shrink) [5]) using artificial neural networks for base forecasts ${\hat{y}}_{t}$
**NN+SR**(λ_1_, λ_*M*_): our structured regularization model (10) applied to artificial neural networks with regularization parameters λ_1_ and λ_*M*_; see also Eq (11)

Here, we determined parameter values for *n* and *α* that minimized RMSE in the training period. During the training period, we tuned regularization parameters λ_1_ and λ_*M*_ through hold-out validation [42].

**Fig 4:**
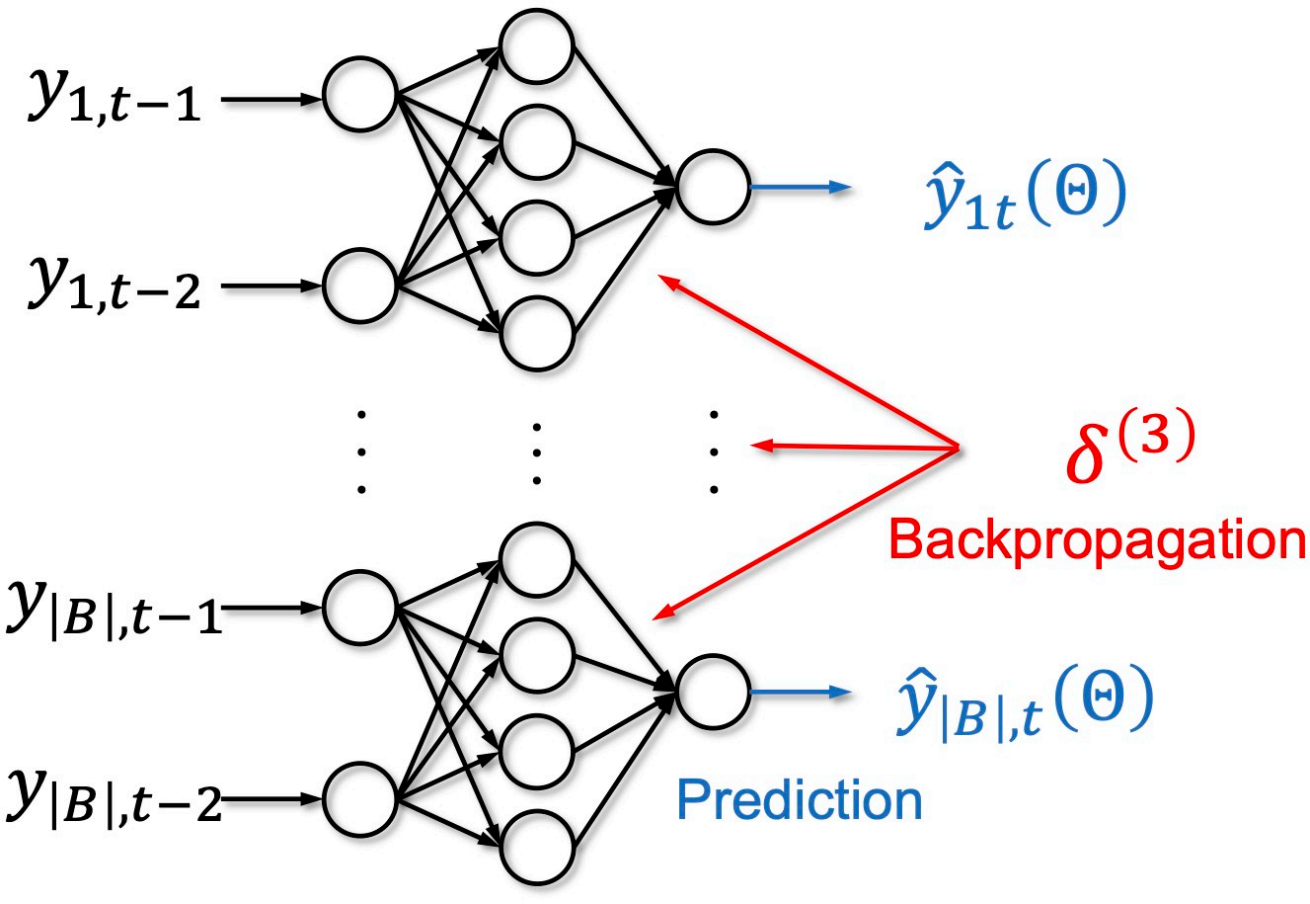
Two-layer neural network adopted in experimental results.

We adopted two-layer artificial neural networks (Fig 4) for NN+BU, NN+MinT, and NN+SR. Here, prediction ${\hat{y}}_{it}\left(\Theta \right)$ of each time series depends on its own two lags *y*_*i*,*t*−1_ and *y*_*i*,*t*−2_, and the backpropagation simultaneously updates weight parameters of all the series. Note also that NN+BU is equivalent to NN+SR(0,0). Following prior studies [43, 44], we set the number of hidden units to twice the number of input units (i.e., |*D*| = 4 ⋅ |*B*|). Bias parameters were added to hidden and output units.

We implemented the backpropagation algorithm (Algorithm 1) in the R programming language, with the convergence threshold set as *ε* = 5 ⋅ 10^−5^. The step size was kept constant and set as *η* = 1 ⋅ 10^−5^, which was small enough for the backpropagation algorithm to converge. We employ the logistic sigmoid function (13) as an activation function. The algorithm was repeated 30 times by randomly generating initial values for the parameter **Θ** from a standard normal distribution. The following sections show average RMSE values with 95% confidence intervals over the 30 trials.

### Synthetic datasets

We generated common factors to express correlations among time series. Denote as *N*(*μ*, *σ*^2^) a normal distribution with mean *μ* and standard deviation *σ*. For common factors, we used the first-order autoregressive models

$$\psi_{it}∼\text{N}\left(\phi_{i}\psi_{i,t-1},\sigma_{i}^{2}\right)\;\left(i\in \left\{1\right\}\cup M,\;t\in T\right),$$

where *ϕ*_*i*_ is an autoregressive coefficient, and *σ*_*i*_ is the standard deviation of white noise for the *i*th common factor. Note that *ψ*_*it*_ reflects the overall trend for *i* = 1 and mid-level trends for *i* ∈ *M* = {2, 3, 4}.

Bottom-level time series were produced by combining the overall trend, mid-level trends, and autocorrelation. We denote the parent (mid-level) node of node *i* as

$$m\left(i\right)=\{\begin{array}{cc} \;2 & (i\in \{5,6,7\}),\\ \;3 & (i\in \{8,9,10\}),\\ \;4 & (i\in \{11,12,13\}). \end{array}$$

For bottom-level time series, we used the first-order autoregressive models

$$y_{it}∼\text{N}\left(\rho_{i}\psi_{1t}+\theta_{i}\psi_{m(i),t}+\phi_{i}y_{i,t-1},\sigma_{i}^{2}\right)\;\left(i\in B,\;t\in T\right),$$

where *ρ*_*i*_ and *θ*_*i*_ respectively indicate effects of the common factors *ψ*_1*t*_ and *ψ*_*m*(*i*), *t*_ on the *i*th time series. After that, we generated upper-level time series (*y*_*it*_ for *i* ∈ *N*\*B*) according to the aggregation constraint (2).

We prepared three synthetic datasets: NgtvC, WeakC, and PstvC. Table 1 lists the parameter values used to generate these datasets. Time series are negatively correlated in the NgtvC dataset, weakly correlated in the WeakC dataset, and positively correlated in the PstvC dataset. Each dataset consists of time series data at 100 time points; the first 70 and latter 30 times were used as training and test periods, respectively.

**Table 1:** Parameter values for the synthetic datasets.

			NgtvC		WeakC		PstvC	
Node *i*	*ϕ*_*i*_	*σ*_*i*_	*ρ*_*i*_	*θ*_*i*_	*ρ*_*i*_	*θ*_*i*_	*ρ*_*i*_	*θ*_*i*_
1	0.3	0.3	—	—	—	—	—	—
2	0.3	0.3	—	—	—	—	—	—
3	0.3	0.3	—	—	—	—	—	—
4	0.3	0.3	—	—	—	—	—	—
5	0.3	0.3	0.1	1.0	0.1	0.1	1.0	1.0
6	0.3	0.3	−0.1	−1.0	0.1	0.1	1.0	1.0
7	0.3	0.3	1.0	0.1	0.1	0.1	1.0	1.0
8	0.3	0.3	0.1	1.0	0.1	0.1	1.0	1.0
9	0.3	0.3	−0.1	−1.0	0.1	0.1	1.0	1.0
10	0.3	0.3	−1.0	0.1	0.1	0.1	1.0	1.0
11	0.3	0.3	0.1	1.0	0.1	0.1	1.0	1.0
12	0.3	0.3	−0.1	−1.0	0.1	0.1	1.0	1.0
13	0.3	0.3	1.0	0.1	0.1	0.1	1.0	1.0

We standardized the generated time series according to the mean and variance over the training period when using artificial neural networks. Specifically, we standardized each bottom-level time series for NN+BU and NN+SR and summed them appropriately to calculate upper-level time series for NN+SR. We standardized each time series at all levels for NN+MinT. We then computed predictive values for these time series and transformed the obtained predictive values into the original (unstandardized) scale. After that, we applied the bottom-up method (NN+BU and NN+SR) and the forecast reconciliation method (NN+MinT) to make coherent forecasts. Finally, we calculated RMSEs on the original scale to evaluate prediction performance.

### Results for synthetic datasets

Tables 2–4 list the out-of-sample RMSE values provided by each method for each node in the NgtvC, WeakC, and PstvC datasets. In the tables, the rows labeled “Mid-level” and “Bottom-level” show the average RMSE values over the mid- and bottom-level nodes, respectively, with smallest RMSE values for each node indicated in bold. Note that average RMSE values with 95% confidence intervals are shown for NN+BU, NN+MinT, and NN+SR.

**Table 2:** Prediction performance for the NgtvC dataset.

	RMSE				
Node *i*	MA(12)	ES(0.20)	NN+BU	NN+MinT	NN+SR(0.0, 2.1)
Root	**1.09**	1.10	1.16 ± 0.04	**1.09** ± 0.01	1.15 ± 0.01
2	0.63	0.64	0.66 ± 0.03	**0.60** ± 0.01	**0.60** ± 0.01
3	0.80	0.76	0.76 ± 0.01	**0.73** ± 0.01	**0.73** ± 0.01
4	0.71	0.72	0.70 ± 0.02	0.69 ± 0.01	**0.67** ± 0.01
Mid-level	0.71	0.71	0.71 ± 0.01	**0.67** ± 0.01	**0.67** ± 0.01
5	0.53	0.48	0.48 ± 0.02	0.47 ± 0.02	**0.44** ± 0.01
6	0.67	**0.64**	0.65 ± 0.02	**0.64** ± 0.01	**0.64** ± 0.02
7	0.39	**0.37**	0.38 ± 0.00	0.39 ± 0.01	0.38 ± 0.00
8	0.42	**0.39**	0.41 ± 0.01	0.41 ± 0.01	0.41 ± 0.01
9	**0.35**	**0.35**	0.38 ± 0.01	0.38 ± 0.01	0.38 ± 0.01
10	0.58	0.56	0.55 ± 0.02	**0.53** ± 0.01	**0.53** ± 0.01
11	0.58	0.49	**0.47** ± 0.01	**0.47** ± 0.01	**0.47** ± 0.01
12	0.50	0.47	0.47 ± 0.01	**0.46** ± 0.01	**0.46** ± 0.00
13	0.48	0.48	0.47 ± 0.01	0.47 ± 0.01	**0.46** ± 0.01
Bottom-level	0.50	0.47	0.47 ± 0.00	0.47 ± 0.00	**0.46** ± 0.00
Average	0.60	0.57	0.58 ± 0.00	**0.56** ± 0.00	**0.56** ± 0.00

**Table 3:** Prediction performance for the WeakC dataset.

	RMSE				
Node *i*	MA(12)	ES(0.00)	NN+BU	NN+MinT	NN+SR(0.0, 1.2)
Root	1.06	**1.00**	1.06 ± 0.02	1.05 ± 0.01	1.06 ± 0.01
2	0.46	**0.41**	0.45 ± 0.01	0.43 ± 0.01	0.44 ± 0.01
3	0.60	**0.56**	0.60 ± 0.01	0.58 ± 0.01	0.58 ± 0.01
4	0.61	0.60	0.59 ± 0.01	0.58 ± 0.01	**0.57** ± 0.01
Mid-level	0.56	**0.52**	0.55 ± 0.00	0.53 ± 0.01	0.53 ± 0.00
5	0.32	**0.30**	0.31 ± 0.01	0.31 ± 0.01	**0.30** ± 0.00
6	0.39	**0.37**	0.39 ± 0.01	0.38 ± 0.01	0.38 ± 0.01
7	**0.24**	**0.24**	0.25 ± 0.00	0.25 ± 0.01	**0.24** ± 0.00
8	0.30	**0.29**	0.33 ± 0.01	0.32 ± 0.01	0.31 ± 0.01
9	0.27	**0.26**	0.27 ± 0.01	0.27 ± 0.01	**0.26** ± 0.01
10	0.37	**0.34**	0.35 ± 0.01	**0.34** ± 0.01	0.35 ± 0.01
11	0.39	0.36	0.34 ± 0.01	0.34 ± 0.01	**0.33** ± 0.01
12	0.37	**0.36**	0.37 ± 0.01	**0.36** ± 0.01	**0.36** ± 0.01
13	0.29	0.29	0.29 ± 0.00	0.29 ± 0.01	**0.28** ± 0.00
Bottom-level	0.33	**0.31**	0.32 ± 0.00	0.32 ± 0.00	**0.31** ± 0.00
Average	0.44	**0.41**	0.43 ± 0.00	0.42 ± 0.00	0.42 ± 0.00

**Table 4:** Prediction performance for the PstvC dataset.

	RMSE				
Node *i*	MA(1)	ES(0.89)	NN+BU	NN+MinT	NN+SR(0.4, 2.4)
Root	2.69	2.69	2.90 ± 0.05	2.58 ± 0.07	**2.49** ± 0.03
2	1.20	1.20	1.33 ± 0.03	1.16 ± 0.03	**1.12** ± 0.01
3	1.49	1.42	**1.12** ± 0.01	1.16 ± 0.03	1.27 ± 0.02
4	1.11	1.11	1.25 ± 0.03	1.63 ± 0.02	**1.06** ± 0.01
Mid-level	1.27	1.24	1.23 ± 0.01	1.32 ± 0.03	**1.15** ± 0.01
5	0.53	0.53	0.56 ± 0.01	**0.50** ± 0.01	0.53 ± 0.01
6	0.49	0.49	0.55 ± 0.02	**0.48** ± 0.01	0.51 ± 0.02
7	0.46	0.46	0.49 ± 0.01	0.46 ± 0.01	**0.43** ± 0.01
8	0.56	0.55	**0.48** ± 0.01	**0.48** ± 0.01	0.54 ± 0.03
9	0.57	0.54	**0.42** ± 0.01	0.46 ± 0.02	0.53 ± 0.04
10	0.49	0.47	0.43 ± 0.01	**0.42** ± 0.01	0.45 ± 0.01
11	**0.48**	**0.48**	0.55 ± 0.02	0.51 ± 0.01	0.49 ± 0.02
12	0.59	0.57	0.51 ± 0.01	**0.48** ± 0.01	0.55 ± 0.02
13	0.44	0.44	0.45 ± 0.02	**0.42** ± 0.01	0.43 ± 0.01
Bottom-level	0.51	0.50	0.49 ± 0.00	**0.47** ± 0.01	0.50 ± 0.00
Average	0.85	0.84	0.85 ± 0.00	0.83 ± 0.01	**0.80** ± 0.00

For the NgtvC dataset (Table 2), our structured regularization method NN+SR was comparable to the forecast reconciliation method and outperformed the other methods, except for the RMSE of the root node. For the WeakC dataset (Table 3), our method was slightly inferior to the exponential smoothing method, but the differences were minimal. For the PstvC dataset (Table 4), our method attained the best prediction performance in terms of average RMSE. These results show that our structured regularization method delivered good prediction performance for the three synthetic datasets. Our method was especially effective when the time series were strongly correlated, as in the NgtvC and PstvC datasets.

We next focus on the parameter values for our structured regularization. Only for the PstvC dataset, our method NN+SR(λ_1_, λ_*M*_) adopted λ_1_ > 0 and performed significantly better than the bottom-up method in terms of the RMSE of the root node. Additionally, our method employed λ_*M*_ > 0 for all three datasets and outperformed the bottom-up method for mid-level RMSEs. These results show an association between regularization weights and prediction accuracy at each time series level. Our method adjusts the regularization parameters to fit the data characteristic, thereby achieving better prediction performance.

**Fig 5:**
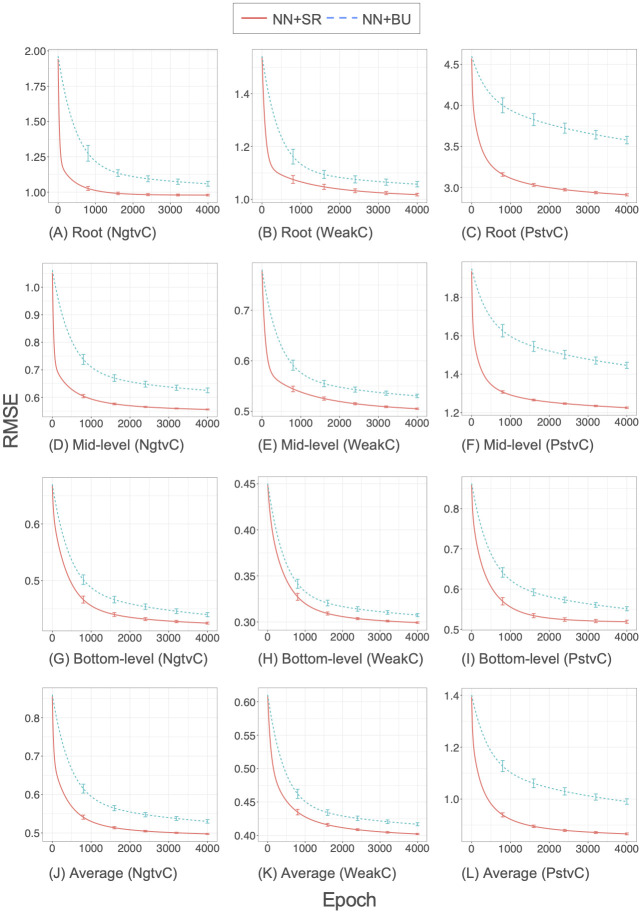
Convergence of the backpropagation algorithm for the synthetic datasets.

Fig 5 shows the training RMSE values as a function of the epoch (number of iterations) in the backpropagation algorithm for the synthetic datasets. Note that the computational efficiency can be evaluated based on epochs because little difference existed between NN+SR and NN+BU in the computation time required for one epoch.

RMSEs decreased faster for our structured regularization method NN+SR than for the bottom-up method NN+BU. The convergence performance of the two methods greatly differed, especially for the PstvC dataset and upper-level time series. Consequently, our structured regularization method improved both prediction accuracy and convergence speed of the backpropagation algorithm. This suggests that our method will deliver good prediction performance even if the backpropagation algorithm is terminated in the middle of computation.

**Fig 6:**
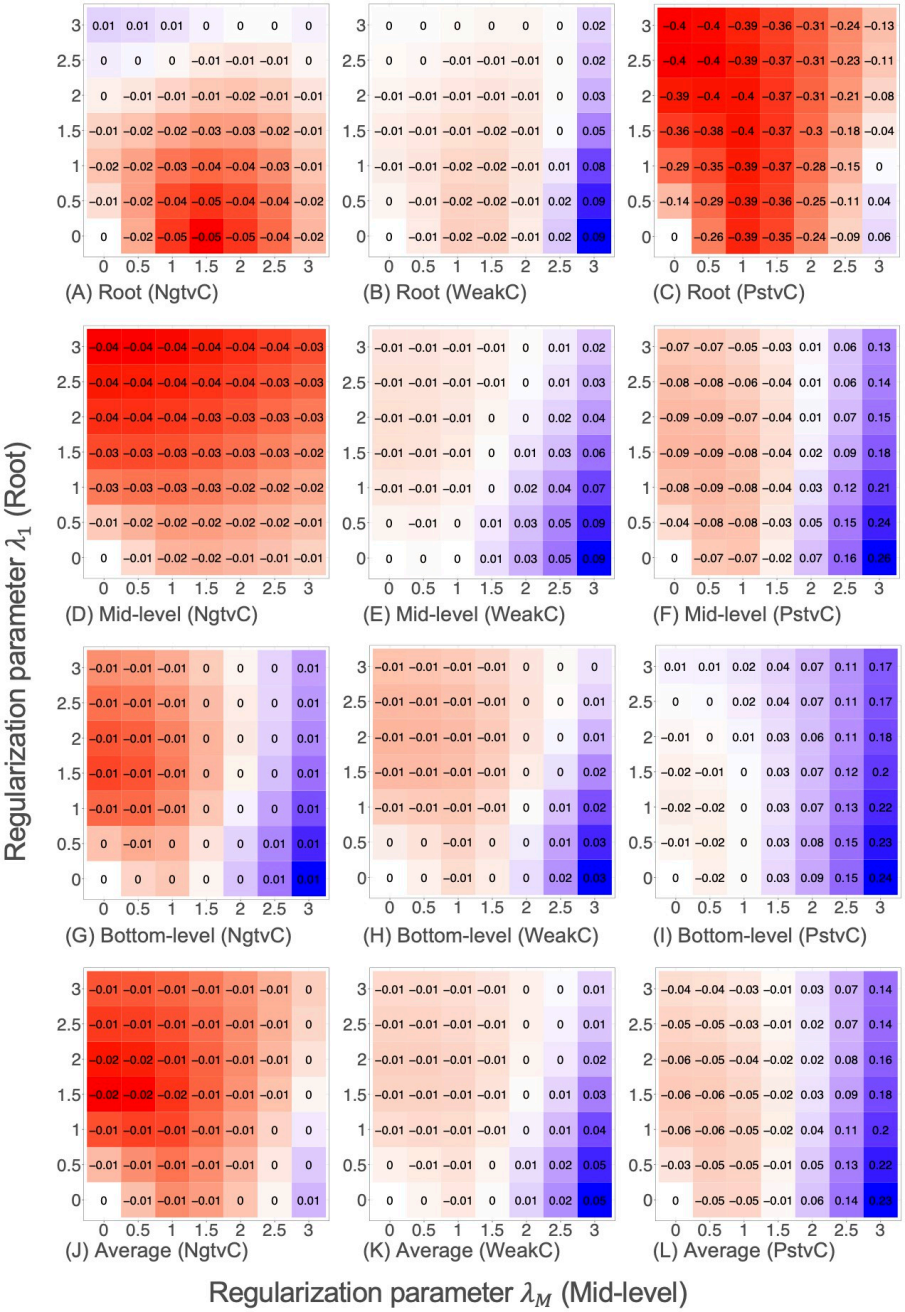
Heat maps of relative RMSEs provided by NN+SR(λ_1_, λ_*M*_) in the synthetic datasets.

Fig 6 shows heat maps of the out-of-sample relative RMSE values provided by our structured regularization method NN+SR(λ_1_, λ_*M*_) for the synthetic datasets. Here, the vertical and horizontal axes are the values of regularization parameters λ_1_ and λ_*M*_, respectively. This figure shows how regularization for each time series level affects the prediction performance.

Note that RMSE values were normalized in Fig 6 such that the RMSE for (λ_1_, λ_*M*_) = (0, 0) was zero (white-colored) in each trial. Accordingly, the corresponding regularization is effective if the relative RMSE value is negative (red-colored), and it is ineffective if the relative RMSE value is positive (blue-colored). RMSEs were consistently reduced in the NgtvC dataset. Regularization was particularly effective for the root time series in the PstvC dataset. RMSE values tend to vary drastically from left to right in each heat map, which suggests that the regularization for the mid-level time series greatly impacted prediction performance.

### Real-world datasets

We downloaded historical data describing unemployment rates in Japan from e-Stat, a portal site for official Japanese statistics (https://www.e-stat.go.jp/en). Using these data, we prepared three real-world datasets for Japanese regions: Tohoku, Chubu, and Kansai. Table 5 lists the prefectures forming the two-level hierarchical structure (Fig 3).

**Table 5:** List of prefectures in the real-world datasets.

	Prefectures		
Node *i*	Tohoku	Chubu	Kanasi
5	Aomori	Niigata	Mie
6	Iwate	Toyama	Shiga
7	Miyagi	Ishikawa	Kyoto
8	Akita	Fukui	Osaka
9	Yamagata	Yamanashi	Hyogo
10	Fukushima	Nagano	Nara
11	Ibaraki	Gifu	Wakayama
12	Tochigi	Shizuoka	Tottori
13	Gunma	Mie	Okayama

We used quarterly statistics (model-based estimates) of unemployment rates during 90 time periods from January 1997 to June 2019, taking the first 60 and last 30 time periods as the training and test periods, respectively. As a preprocessing step, we removed seasonal and trend components by means of the stl function in the R stats package. We next calculated upper-level time series according to the aggregation constraint (2). After that, we standardized time series, computed predicted values, and calculated RMSEs on the original scale, in the same way as for the synthetic datasets.

### Results for real-world datasets

Tables 6–8 list the out-of-sample RMSE values provided by each method for each node in the Tohoku, Chubu, and Kansai datasets. For the Tohoku dataset (Table 6), our structured regularization method NN+SR was comparable to the forecast reconciliation method and substantially outperformed the other methods. For the Chubu dataset (Table 7), our method attained the second-best value for average RMSE.

**Table 6:** Prediction performance for the Tohoku dataset.

	RMSE				
Node *i*	MA(20)	ES(0.12)	NN+BU	NN+MinT	NN+SR(0.4, 1.5)
Root	6.32	6.45	5.98 ± 0.07	5.87 ± 0.06	**5.70** ± 0.06
2	2.83	2.91	2.77 ± 0.05	**2.65** ± 0.05	2.66 ± 0.03
3	2.06	2.13	2.02 ± 0.05	2.04 ± 0.04	**2.01** ± 0.03
4	2.86	2.92	2.70 ± 0.04	2.68 ± 0.03	**2.63** ± 0.01
Mid-level	2.58	2.65	2.50 ± 0.01	2.46 ± 0.03	**2.43** ± 0.01
5	1.69	1.76	1.68 ± 0.06	**1.59** ± 0.04	1.65 ± 0.05
6	0.76	0.77	0.77 ± 0.03	**0.70** ± 0.02	0.72 ± 0.02
7	1.15	1.17	1.14 ± 0.04	1.12 ± 0.03	**1.11** ± 0.03
8	0.79	0.82	0.79 ± 0.03	0.76 ± 0.03	**0.74** ± 0.02
9	0.88	0.91	0.86 ± 0.03	0.87 ± 0.02	**0.83** ± 0.03
10	1.01	1.04	1.01 ± 0.03	**1.00** ± 0.03	**1.00** ± 0.03
11	1.21	1.24	1.21 ± 0.03	**1.19** ± 0.02	1.25 ± 0.04
12	0.90	0.92	**0.88** ± 0.03	**0.88** ± 0.01	0.89 ± 0.02
13	0.98	1.00	0.94 ± 0.02	**0.88** ± 0.03	0.94 ± 0.02
Bottom-level	1.04	1.07	1.03 ± 0.01	**1.00** ± 0.01	1.01 ± 0.00
Average	1.80	1.85	1.75 ± 0.01	1.71 ± 0.02	**1.70** ± 0.01

**Table 7:** Prediction performance for the Chubu dataset.

	RMSE				
Node *i*	MA(16)	ES(0.03)	NN+BU	NN+MinT	NN+SR(0.4, 0.6)
Root	4.11	4.09	3.99 ± 0.04	4.00 ± 0.04	**3.97** ± 0.03
2	1.77	1.75	1.72 ± 0.03	**1.71** ± 0.03	1.72 ± 0.03
3	1.38	1.37	1.37 ± 0.03	**1.35** ± 0.02	1.36 ± 0.03
4	2.19	2.17	2.18 ± 0.03	**2.15** ± 0.03	2.18 ± 0.03
Mid-level	1.78	1.76	1.76 ± 0.01	**1.74** ± 0.02	1.75 ± 0.01
5	0.80	0.79	0.81 ± 0.03	**0.78** ± 0.02	0.81 ± 0.03
6	0.67	0.65	0.65 ± 0.03	**0.64** ± 0.02	0.66 ± 0.03
7	0.76	0.76	**0.74** ± 0.03	**0.74** ± 0.02	**0.74** ± 0.03
8	0.82	0.81	0.77 ± 0.02	0.77 ± 0.03	**0.76** ± 0.02
9	0.71	0.70	0.68 ± 0.02	0.69 ± 0.02	**0.67** ± 0.02
10	0.95	**0.97**	0.99 ± 0.02	**0.97** ± 0.02	0.98 ± 0.02
11	0.81	**0.80**	0.88 ± 0.04	0.86 ± 0.03	0.89 ± 0.04
12	0.99	0.98	0.98 ± 0.03	**0.96** ± 0.02	0.97 ± 0.03
13	**0.73**	**0.73**	0.75 ± 0.02	0.75 ± 0.02	0.77 ± 0.02
Bottom-level	**0.80**	**0.80**	0.81 ± 0.00	**0.80** ± 0.01	0.81 ± 0.00
Average	1.28	1.27	1.27 ± 0.00	**1.26** ± 0.01	1.27 ± 0.00

**Table 8:** Prediction performance for the Kansai dataset.

	RMSE				
Node *i*	MA(18)	ES(0.05)	NN+BU	NN+MinT	NN+SR(0.4, 1.2)
Root	13.88	13.84	13.60 ± 0.68	12.47 ± 0.30	**12.20** ± 0.39
2	2.57	2.56	2.58 ± 0.09	2.43 ± 0.05	**2.37** ± 0.04
3	12.78	12.79	12.56 ± 0.69	11.57 ± 0.30	**11.14** ± 0.41
4	1.90	1.90	1.83 ± 0.04	1.81 ± 0.04	**1.68** ± 0.03
Mid-level	5.75	5.75	5.66 ± 0.12	5.27 ± 0.11	**5.06** ± 0.07
5	0.73	0.74	0.77 ± 0.03	**0.72** ± 0.02	0.79 ± 0.02
6	1.80	1.82	1.87 ± 0.07	**1.77** ± 0.04	1.83 ± 0.04
7	1.35	1.36	1.33 ± 0.07	1.35 ± 0.05	**1.22** ± 0.04
8	11.31	11.34	11.29 ± 0.66	10.38 ± 0.27	**10.02** ± 0.39
9	2.71	2.69	2.62 ± 0.14	2.60 ± 0.12	**2.43** ± 0.10
10	1.50	1.49	1.48 ± 0.07	1.45 ± 0.06	**1.43** ± 0.06
11	1.16	1.14	1.14 ± 0.04	1.12 ± 0.04	**1.03** ± 0.03
12	0.82	0.82	0.79 ± 0.02	0.81 ± 0.01	**0.78** ± 0.01
13	0.99	0.99	0.96 ± 0.03	0.96 ± 0.03	**0.95** ± 0.02
Bottom-level	2.49	2.49	2.47 ± 0.04	2.35 ± 0.04	**2.28** ± 0.02
Average	4.12	4.11	4.06 ± 0.08	3.80 ± 0.07	**3.68** ± 0.05

For the Kansai dataset (Table 8), our method greatly exceeded the prediction performance of the other methods. These results demonstrate that our structured regularization method achieved good prediction performance for the three real-world datasets.

**Fig 7:**
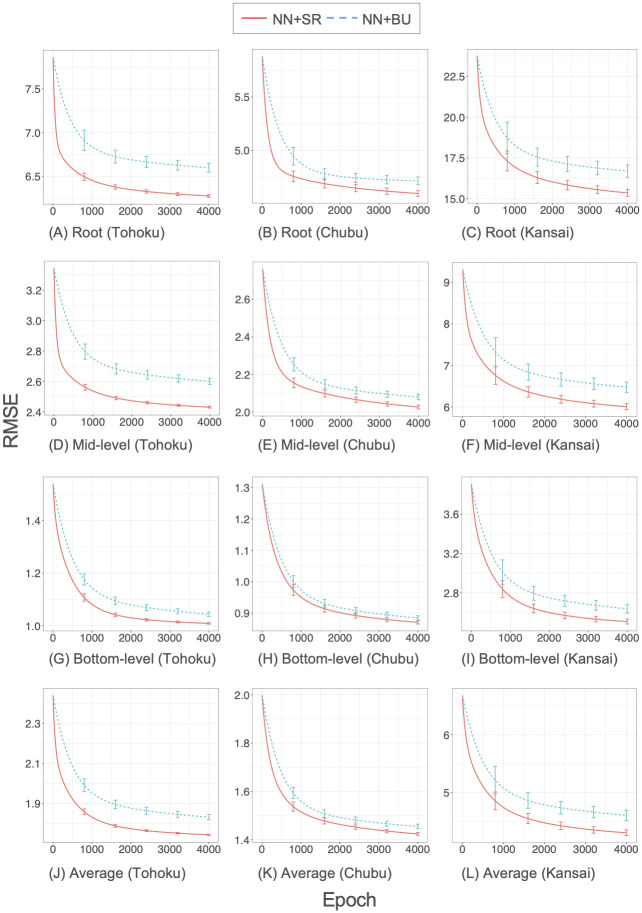
Convergence of the backpropagation algorithm for the real-world datasets.

Fig 7 shows the training RMSE values as a function of epoch in the backpropagation algorithm for the real-world datasets. The convergence of RMSE values was consistently faster for our structured regularization method NN+SR than for the bottom-up method NN+BU. For the Tohoku and Chubu datasets, our method greatly accelerated convergence for upper-level time series. For the Kansai dataset, our method was superior to the bottom-up method in terms of both prediction accuracy and convergence speed. These results suggest that our structured regularization method improves the convergence performance of the backpropagation algorithm.

**Fig 8:**
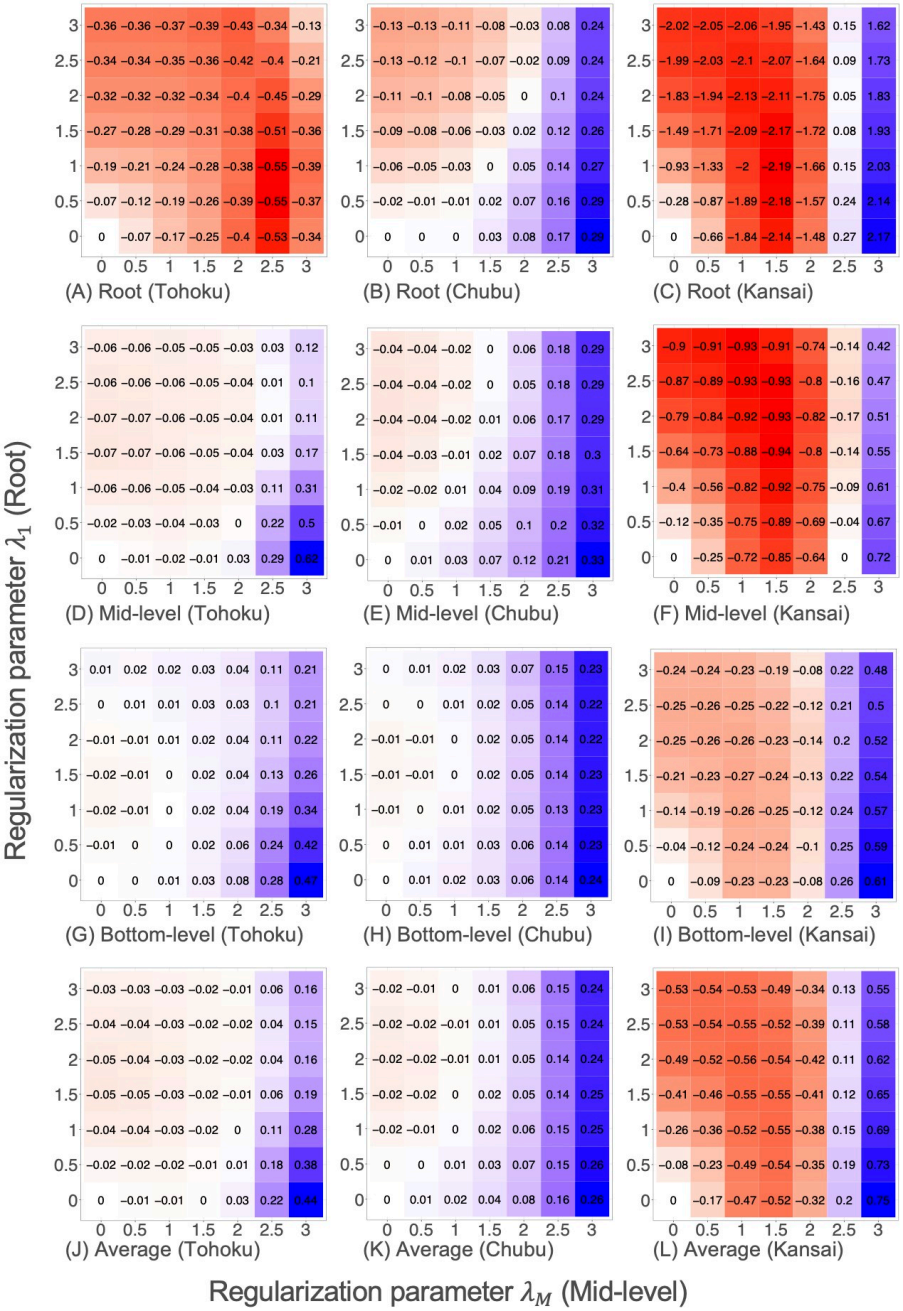
Heat maps of relative RMSEs provided by NN+SR(λ_1_, λ_*M*_) in the real-world datasets.

Fig 8 shows heat maps of the out-of-sample relative RMSE values provided by our structured regularization method NN+SR(λ_1_, λ_*M*_) for the real-world datasets. For the Tohoku dataset, the reduction in RMSE values was particularly large for the root time series. For the Chubu dataset, RMSE values changed greatly from left to right in each heat map, meaning that the regularization for mid-level time series was the most effective. For the Kansai dataset, RMSE values can be reduced greatly for all time series levels if the regularization parameters are properly tuned.

## Conclusion

We proposed a structured regularization model for predicting hierarchical time series. Our model uses the regularization term for improving upper-level forecasts to correct bottom-level forecasts. We demonstrated the application of our model to artificial neural networks for time series prediction. We also developed a backpropagation algorithm specialized for training our model based on artificial neural networks.

We investigated the efficacy of our method through experiments using synthetic and real-world datasets. The experimental results demonstrated that our method, which can adjust regularization parameters to fit data characteristics, compared favorably in prediction performance with other methods that develop coherent forecasts for hierarchical time series. Our regularization term accelerated the backpropagation algorithm. Regularization for mid-level time series was closely related to prediction performance.

One contribution made by this study is the establishment of a new computational framework of artificial neural networks for time series predictions. Moreover, our experiments using synthetic and real-world datasets demonstrated the potential of specialized prediction methods for hierarchical time series. We hope that this study will stimulate further research on exploiting structural properties for better time series predictions.

In future studies, we will extend our structured regularization model to other time series prediction methods, such as the autoregressive integrated moving average model [6, 7] and support vector regression [8]. Another direction of future research will be to develop a high-performance estimation algorithm for our method based on various mathematical optimization techniques [45–50].
